# The efficacy and safety of high versus low doses of intravenous intraoperative tranexamic acid during spinal fusion in patients with adolescent idiopathic scoliosis: a network meta-analysis of randomized controlled trials

**DOI:** 10.1007/s43390-026-01289-y

**Published:** 2026-01-24

**Authors:** Omkar S. Anaspure, Anthony N. Baumann, Grayson M. Talaski, Mark Villers, Keith D. Baldwin

**Affiliations:** 1 Perelman School of Medicine, University of Pennsylvania, 3400 Civic Center Blvd, Philadelphia, PA 19140 USA; 2 Department of Orthopaedic Surgery, Cleveland Clinic Akron General, Akron, OH USA; 3 Department of Mechanical Engineering, University of Iowa, Iowa City, IA USA; 4 College of Medicine, Northeast Ohio Medical University, Rootstown, OH USA; 5 Department of Orthopaedic Surgery, Children’s Hospital of Philadelphia, Philadelphia, PA USA

**Keywords:** Adolescent idiopathic scoliosis, Tranexamic acid, Posterior spinal fusion, Dose-dependent

## Abstract

**Introduction:**

Posterior spinal fusion (PSF) for adolescent idiopathic scoliosis entails notable bleeding and transfusion risk, yet optimal tranexamic acid dosing remains undefined. We conducted a network meta-analysis (NMA) comparing high dose, low dose, and no-TXA regimens in this setting.

**Methods:**

We conducted a PROSPERO-registered NMA (CRD420251033929) of RCTs in AIS patients undergoing PSF. PubMed, CINAHL, EMBASE, reference lists, and grey literature were searched for trials comparing high dose (20–50 mg/kg load; 10–20 mg/kg/h infusion), low dose (10 mg/kg load; 1 mg/kg/h infusion), or no TXA. Outcomes were pooled using a random-effects model to produce mean differences for continuous data and relative risks for binary data.

**Results:**

Five RCTs (n = 475) were included. Patients had a frequency‐weighted average (FWA) (SD) age of 15.1 (1.5) years, preoperative Cobb angle of 58.0 (7.9)°, 10.4 (1.3) levels fused, and operative time of 186.9 (62.0) minutes. They were allocated to high‐dose TXA (n = 184), low‐dose TXA (n = 144), or no‐TXA (n = 147) arms. The FWA total EBL was 787.3 (261.5) mL in the high‐dose group, 705.3 (219.0) mL in the low‐dose group, and 1016.3 (492.2) mL in controls. There was no significant difference in EBL between high‐ vs low‐dose TXA (MD −98.3 mL [−646.9, 426.2]). In the NMA, high‐dose TXA reduced total EBL by 319 mL (95% CI −818 to 133) and low‐dose by 219 mL (95% CI −764 to 294) versus no TXA—an 81% probability that no TXA was worst strategy—though neither comparison reached statistical significance. When compared per fused level, High‐ and low‐dose TXA reduced EBL per level by 38.2 mL (MD −38.2 [−86.3, 6.1]) and 29.5 mL (MD −29.5 [−85.2, 27.3]) versus no TXA, respectively, without statistical significance; however, the no‐TXA arm had an 87% probability of being worst for EBL by level. The FWA EBL per level was 78.9 ± 6.3 mL, 78.2 ± 7.2 mL, and 116.3 ± 17.8 mL for high‐dose, low‐dose, and no‐TXA groups (very low certainty). When compared by operative time, high and lowdose TXA reduced EBL per hour by 81.0 mL/h (MD −81.0 [−250.0, 80.5]) and 60.2 mL/h (MD −60.2 [−285.0, 160.0]) versus no TXA, respectively, with no statistical significance. FWA EBL per hour was 273.8 ± 112.6 mL/h, 315.4 ± 133.6 mL/h, and 249.8 ± 150.2 mL/h for high dose, low dose, and no TXA (very low certainty). Both TXA arms had no complications vs. one uncontrolled bleed in the no-TXA group (0.7%).

**Conclusion:**

High and low dose TXA in AIS PSF yielded modest, non-significant reductions in total blood loss and per-level EBL. No thromboembolic, neurologic, or renal complications occurred among, underscoring its safety. These results support a case-by-case approach to TXA use and highlight the need for larger, standardized RCTs to confirm its clinical value.

**Supplementary Information:**

The online version contains supplementary material available at 10.1007/s43390-026-01289-y.

## Introduction

Adolescent idiopathic scoliosis (AIS) is a pediatric spinal deformity that emerges during puberty and affects 1–3% of adolescents, with a higher prevalence in females [1–4]. Bracing is the first-line treatment for curves ≤ 40°, but severe or progressive deformities require surgical correction with posterior spinal fusion (PSF) remaining the gold standard for curves > 40° [5–9]. Despite reliably correcting deformity, PSF is consistently associated with substantial intraoperative hemorrhage—reported mean blood losses range from 275 mL to over 900 mL per procedure due to extensive soft tissue dissection and osteotomies [10–13]**.** Excessive bleeding not only heightens transfusion requirements but also increases risks of anemia, prolonged recovery, and potential cardiopulmonary compromise [14, 15]**.**

Tranexamic acid (TXA) is a synthetic lysine analogue which inhibits fibrinolysis by blocking lysine‐binding sites on plasminogen to stabilize clot formation, gaining popularity as an adjunct for minimizing blood loss in spine surgery [16–18]. Recent randomized controlled trials (RCT) in AIS patients demonstrated that TXA significantly reduces blood loss in a variety of surgical settings, including scoliosis correction. Goobie et al. (2018) reported that highdose TXA (50 mg/kg loading, 10 mg/kg/h maintenance) reduced mean estimated blood loss (EBL) to 836 mL versus 1031 mL in controls, with no thromboembolic events [17]. Similarly, Saleh et al. (2018) found an EBL of 181 mL in high dose TXA (Loading dose: 50 mg/kg; maintenance dose: 20 mg/kg/hour) versus 267 mL EBL without TXA, again noting no increase in complications [19]. Despite encouraging results, TXA’s dose-dependent risks—venous thromboembolisms (VTE), seizures, cardiac complications, and strokes—remain significant concerns for spinal surgeons, prompting investigation into whether lower doses can maintain efficacy while reducing adverse events [16–18]**.**

In response, several systematic reviews and meta-analyses have attempted to synthesize available data. Xiong et al. (2020) pooled six RCTs and eleven non-RCTs (total n = 1148) across various spinal deformities, finding that both high and low dose TXA significantly reduced total and intraoperative blood loss versus placebo, and that high dose was linked to more fused segments but similar complication rates [20]. However, their analysis was confounded by combined AIS with other deformity etiologies and included studies without direct high versus low dose comparisons. Liu et al. (2025), focusing exclusively on AIS, integrated two RCTs and four retrospective studies (n = 611), reporting that highdose TXA significantly reduced intraoperative blood loss and transfusion rates. However, these findings were limited ny using primarily retrospective studies with significant heterogeneity (I^2^ up to 97%) [21]. Aleid et al. (2024) analyzed four AIS trials (n = 531), concluding high dose TXA modestly outperformed low dose in reducing EBL (mean difference − 0.40 L) but found no difference in transfusion or hemoglobin decline; they also highlighted very low certainty of evidence due to limited trial numbers and variable TXA protocols [13].

Collectively, these early trials and meta analyses underscore TXA’s potential to reduce blood loss specifically in AIS surgery, yet leave critical gaps. Direct, well-powered comparisons between high and low dosing remain scarce; existing meta-analyses are hampered by heterogeneity in study design, mixed deformity populations, and underreporting of adverse events. Consequently, optimal TXA dosing to balance efficacy and safety in AIS PSF remains undefined. Our study addresses these gaps by focusing exclusively on AIS patients undergoing PSF and by employing a network meta-analysis (NMA) which enables more robust, direct comparisons across different TXA dosing regimens. Therefore, the purpose of this NMA is to compare the efficacy and safety of high dose versus low dose TXA during spinal fusion for patients with AIS, aiming to better inform decision-making parameters regarding optimal TXA use for bleeding management.

Methods

### Study registration and guideline adherence

This study is a NMA of RCTs examining the efficacy and safety of intraoperative administration of TXA during spinal fusion for patients with AIS. This NMA was pre-registered on the International Prospective Register of Systematic Reviews (PROSPERO) prior to the article sorting process (CRD420251033929). Additionally, this NMA was conducted under the guidance of the Preferred Reporting Items for Systematic Reviews and Meta-Analysis Extension for Network Meta-Analyses (PRISMA-NMA) guidelines to enhance quality and readability [22].

### Study creation with search strategy

This NMA search PubMed, CINAHL, and EMBASE from database inception until April 17th, 2025, using the following search algorithm: (tranexamic acid) AND ("adolescent scoliosis" OR "adolescent idiopathic scoliosis" OR "adolescent scoliosis surgery" OR "pediatric scoliosis surgery" OR pediatric scoliosis surgery) AND ("randomized placebo-controlled trial" OR randomized controlled trial OR "randomized double-blind" OR randomized OR randomized trial OR "double-blinded" OR "double-blind" OR "randomly divided"). Additionally, a gray literature search was conducted for comprehensiveness using the “related articles” feature on Google Scholar to identify any additional articles missed from our initial search in the three databases.

### Inclusion and exclusion criteria

Inclusion criteria included full-text English articles with patients who underwent spinal fusion for surgical correction of AIS, compared dosages of TXA or TXA versus no TXA, had consistent TXA dosages per group, and were randomized controlled trials (RCTs). Included articles could have patients older than 18 years old, as along as they were diagnosed with AIS at some point. Exclusion criteria included articles without full-text, not in English, non-randomized studies, unclear or inconsistent dosages of TXA, non-operative care, or patient diagnoses other than AIS.

### Study group definitions by tranexamic acid dose

For the purposes of this analysis, the “no TXA” group refers to control arms in which patients received either *placebo (saline)* or *no TXA administration*, as reported by the original trial. All such control groups were categorized collectively as “no TXA” to maintain network connectivity and comparability across studies. Patients were stratified into three groups based on dosage of TXA: the High TXA group (loading dose: 20–50 mg/kg; maintenance dose: 10–20 mg/kg/hour), the Low TXA group (loading dose: 10 mg/kg; maintenance dose: 1 mg/kg/hour) and the No TXA group (no TXA given or placebo).

### Article selection process

The article screening process was completed by a single author with the final decision to include being made by the second author. After the initial database search, all retrieved articles were uploaded into Rayyan to facilitate the sorting process [23]. First, duplicate articles were removed and then articles were sorted by title and abstract. Next, articles were sorted by full-text. Upon the final inclusion of any articles, a full reference search of the included articles was completed.

### Data extraction process

Data extraction was completed by a single author with final edits made by the second author. Then, once a final data set was obtained, another author independently verified all of the data for accuracy with any discrepancies resolved by the second author after communication. Data extracted included first author, year of publication, number of patients, patient diagnosis of AIS, dosing parameters for TXA, age, sex, weight, number of levels fused, preoperative Cobb angle, operative time, intraoperative blood loss, transfusion data, laboratory data, and complications.

### Study outcomes

We examined total intraoperative blood loss (estimated or measured) as well as specific outcomes involving intraoperative blood loss by vertebral level and per hour via NMA as an assessment of the efficacy of TXA. Additional outcomes included narrative information on transfusion rates and laboratory values due to data reporting heterogeneity. Finally, we assessed total and specific TXA-related complications via NMA was an assessment of the safety of TXA.

### Article bias assessment

Risk of bias was assessed utilizing the Cochrane Collaboration’s tool for assessing RCTs, in which seven areas of bias were graded as “high risk”, “low risk”, or “unclear” [24]. Bias assessment was done first by a single author and then independently verified by a second author for quality.

### Certainty of evidence

The Grading of Recommendations, Assessment, Development, and Evaluation (GRADE) approach was used to note evidence certainty as “very low”, “low”, “moderate”, or “high” [25].

### Publication bias

As seen elsewhere, publication bias was assessed via searching ClinicalTrials.gov for unpublished studies and investigating study locations and funding sources for the included RCTs [26]. Due to the limited amount of articles included in this NMA, funnel plots were not utilized.

### Transitivity and network geometry

Transitivity between studies was assessed by examining patient age, weight, preoperative Cobb angle, number of fused levels, and operative time. Network geometry was plotted per analysis.

### Statistical synthesis

This NMA utilized the Statistical Package for the Social Sciences (SPSS) version 30.0 for descriptive statistics as well as MetaInsight, an online statistics software, for NMA [27]. For descriptive statistics, frequency weighted average (FWA) was used as baseline demographic variables were presented as means or medians. When possible, medians were converted to means via a online conversion website (https://meta-converter.com/conversions/mean-sd-iqr). For NMA, a random-effects model was used with mean difference (MD) or relative risk (RR) as the effect size for continuous and binary outcomes, respectively. Forest plots and surface under the cumulative ranking curve (SUCRA) were used to compare and rank probability, respectively [28].

## Results

### Search results and article grading

**Fig. 1:**
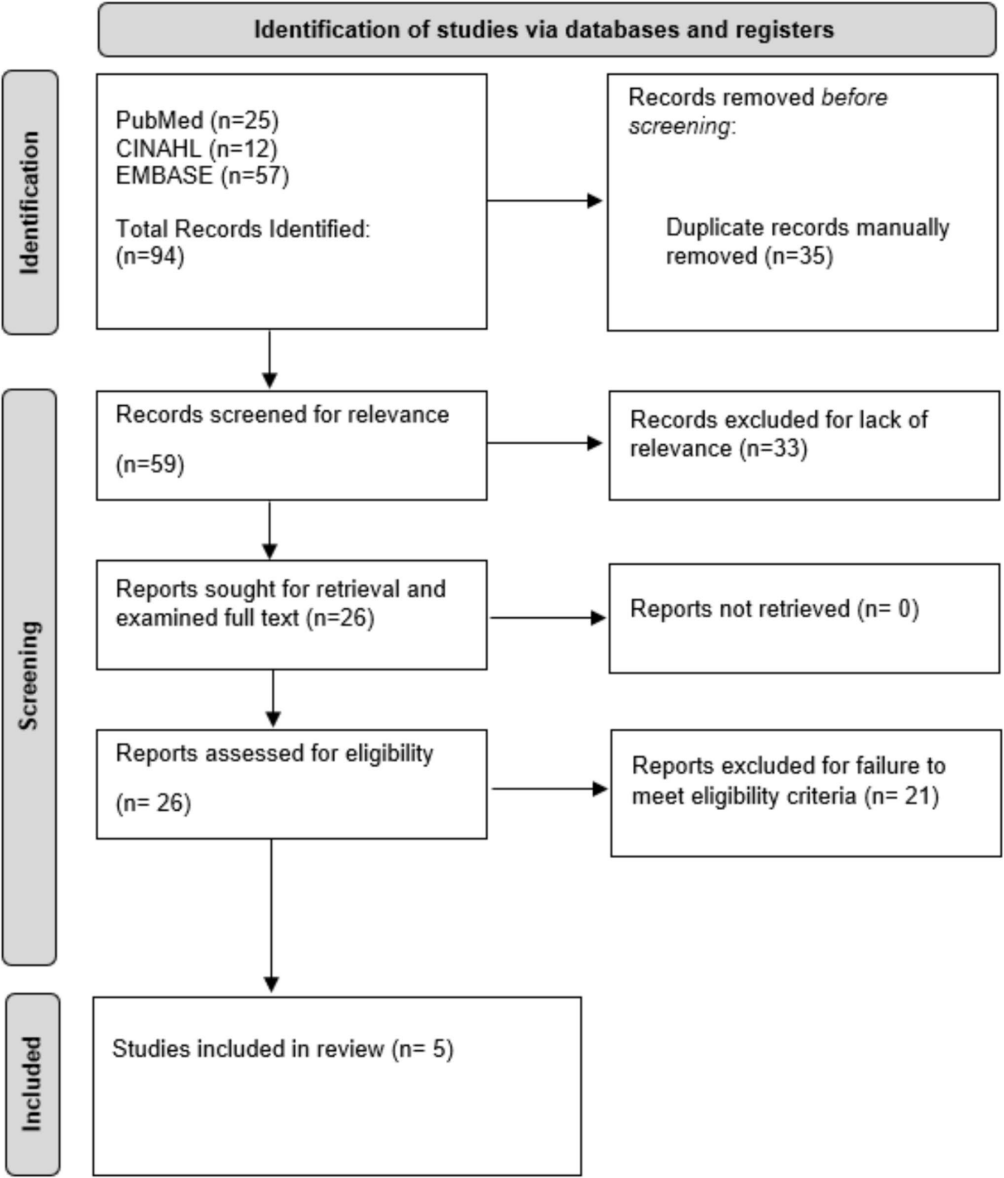
The preferred reporting items for systematic reviews and meta-analyses diagram

Five RCTs were included in this NMA out of 94 articles initially retrieved (Fig. 1) [17–19, 29, 30].

**Fig. 2:**
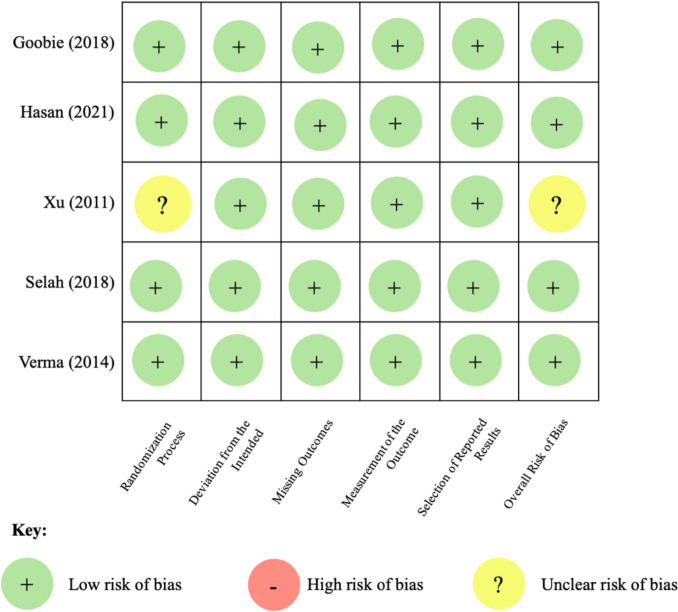
Outcomes of the Cochrane Risk of Bias 2.0 tool for randomized controlled trials; n = 5. The plus sign marks a low risk of bias, and the question mark indicates that there is some concern for bias

For study location, two RCTs (40%) were conducted in the United States with one RCT conducted in either Egypt, Malaysia, or China, respectively. One RCT had funding from the Scoliosis Research Society, three RCTs had no funding, and one RCT did not list funding sources. Furthermore, no additional articles were found on ClinicalTrials.gov, overall indicating a low likelihood of publication bias. All RCTs had low risk of bias, except for Xu et al. (2012), across all risk grading categories (Fig. 2).

### Network structure and connectivity

**Fig. 3:**
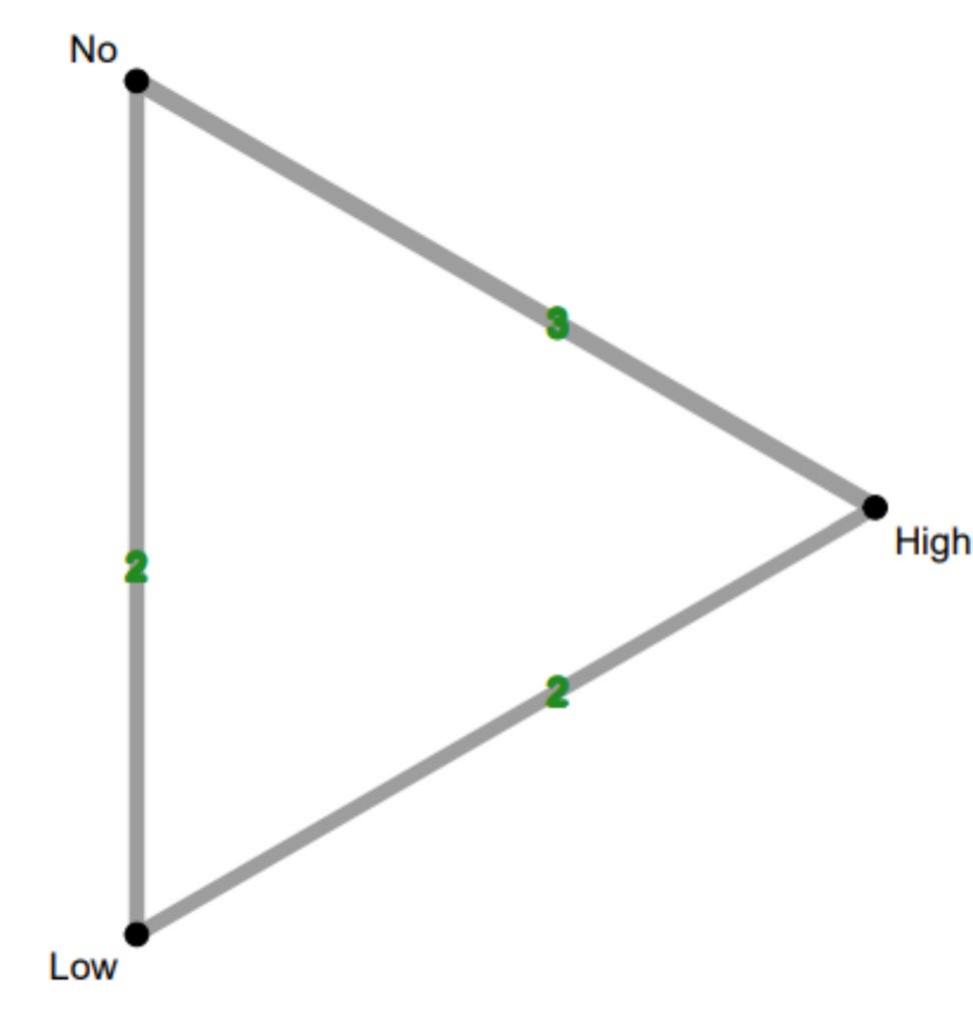
Network geometry plot for total estimated blood loss (EBL) from all included randomized controlled trials (RCTs). Numbers are number of RCTs with direct comparisons

The networks were connected for all our NMA models, including total EBL (Fig. 3) as well as for EBL per level (Supplemental Fig. 1) and EBL per hour (Supplemental Fig. 2).

### Combined patient baseline characteristics

Patients (n = 475) had a FWA age of 15.1 (1.5) years, a FWA weight of 47.9 (6.1) kg, a FWA preoperative Cobb angle of 58.0 (7.9) degrees, a FWA number of levels fused of 10.4 (1.3) levels, and a FWA operative time of 186.9 (62.0) minutes (Table 1). Based on their dosage of TXA, patients were divided into the High TXA group (n = 184), the Low TXA group (n = 144), and the No TXA group (n = 147). For transitivity between the three groups, there was sufficient transitivity for patient age (range of FWA: 14.8–15.6 years; Supplemental Fig. 3), weight (range of FWA: 44.8–51.5 kg; Supplemental Fig. 4), preoperative Cobb angle (range of FWA: 54.2–60.4 degrees; Supplemental Fig. 5), number of fused levels (range of FWA: 9.7–11.0 levels; Supplemental Fig. 6) with variability in operative time (range of FWA: 138.3–245.5 min; Supplemental Fig. 7), prompting sensitivity analyses by level and time.

**Table 1:** Patient baseline characteristics from all five articles

Author (Year)	Study Type	Patient Description	Groups	Patients (n)	Intravenous TXA dosage parameters	Additional TXA information	Patient age (mean (SD)) (years)	Sex (% female)	Weight (mean (SD) or (range)) (kg)	Number of levels fused (median (range) or [IQR] or mean (SD))	Operative time (mean (SD) or median [IQR]) (min)	Cobb angle (median [IQR] or mean (SD)) (degrees)
Goobie (2018)	RCT	AIS	High Dose TXA	56	Loading: 50 mg/kg; maintenance: 10 mg/kg/hour	Loading dose of 50 mg/kg of TXA diluted in normal saline to a volume of 1 mL/kg over 15 min; maintenance dose of 10 mg/kg/hour of 100 mg/kg concentration (0.1 mL/kg/hour)	14.9 (2)	82.14	55.1 (11.8)	10 (5–13)	264 (81)	60 (10)
			No TXA	55	–	–	14.7 (1.8)	76.36	57.6 (11.9)	9 (5–13)	266 (65)	61 (10)
Hasan (2021)	RCT	Patients diagnosed with AIS who were scheduled for elective single-stage posterior spinal fusion	High Dose TXA	83	Loading dose: 30 mg/kg; maintenance dose: 10 mg/kg/hour	IV TXA loading dose given 15 min before incision with maintenance dose until end of surgery; mean total TXA: 2355.7 (554.2)	14.1 (2.1)	91.6	45.3 (9)	11.3 (2.3) Median: 11 [10–13]	130 (37.7) Median: 130 [105–155]	63 [55–82]
			Low Dose TXA	83	Loading dose: 10 mg/kg; maintenance dose: 1 mg/kg/hour	IV TXA loading dose given 15 min before incision with maintenance dose until end of surgery; mean total TXA: 558.5 (113.2)	14.6 (3)	81.9	46.6 (9.3)	11.0 (1.5) Median: 11 [10–12]	123.3 (37.7) Median: 120 [100–150]	64 [57–74]
Saleh (2018)	RCT	Patients with AIS undergoing elective single-stage posterior spine fusion surgery	High Dose TXA	25	Loading dose: 50 mg/kg; maintenance dose: 20 mg/kg/hour	IV TXA loading dose given 30 min before incision with maintenance dose given until skin closure	14.6 (2.07)	40	39.1 (2.5)	–	128.3 (7.6)	43.8 (3.62)
			Low Dose TXA	25	Loading dose: 10 mg/kg; maintenance dose: 1 mg/kg/hour	IV TXA loading dose given 30 min before incision with maintenance dose given until skin closure	14.6 (2.1)	44	39 (3.97)	–	188 (13.41)	43.6 (3.69)
			No TXA	25	–	–	14.6 (2.16)	44	39.4 (4.3)	–	208.3 (21.1)	43.6 (3.69)
Verma (2014)	RCT	Patients with AIS undergoing posterior spinal arthrodesis	Low Dose TXA	36	Loading dose: 10 mg/kg; maintenance dose: 1 mg/kg/hour	IV TXA loading dose infused over 15 min	15.30 (2.37)	88.89	–	8.8 (2.3)	–	54.6 (10.1)
			No TXA	47	–	–	15.01 (2.37)	65.96	–	9 (2.0)	–	54.1 (9.2)
Xu (2012)	RCT	Patients with AIS who underwent spinal fusion for scoliosis correction	High Dose TXA	20	Loading dose: 20 mg/kg; maintenance dose: 10 mg/kg/hour	IV TXA loading dose at skin incision with maintenance dose throughout operation	19.1 (3.2)	40	48.2 (7.4)	12.7 (2.3)	249.2 (49.4)	56.2 (37.4)
			No TXA	20	–	–	20.4 (3.1)	65	49.6 (5.9)	13.1 (1.8)	235.7 (58.1)	48.9 (23.6)

*SD* standard deviation, *RCT* randomized controlled trial, *TXA* tranexamic acid, *IQR* interquartile range

### Patient baseline characteristics by tranexamic acid dose

Patients in the High TXA group had a FWA age of 15.0 (1.5) years, a FWA weight of 47.8 (5.4) kg, a FWA preoperative Cobb angle of 60.4 (7.6) degrees, a FWA number of levels fused of 11.0 (0.9), and a FWA operative time of 183.5 (64.6) minutes. Patients in the Low TXA group had a FWA age of 14.8 (0.3) years, a FWA weight of 44.8 (3.2) kg, a FWA preoperative Cobb angle of 58.7 (8.2) degrees, a FWA number of levels fused of 10.3 (1.0), and a FWA operative time of 138.3 (27.4) minutes. Patients in the No TXA group had a FWA age of 15.6 (1.9) years, a FWA weight of 51.5 (7.6) kg, a FWA preoperative Cobb angle of 54.2 (6.4) degrees, a FWA number of fused levels of 9.7 (1.5) levels, and a FWA operative time of 245.5 (24.5) minutes (Table 1).

### Total estimated blood loss by tranexamic acid dose

**Fig. 4:**
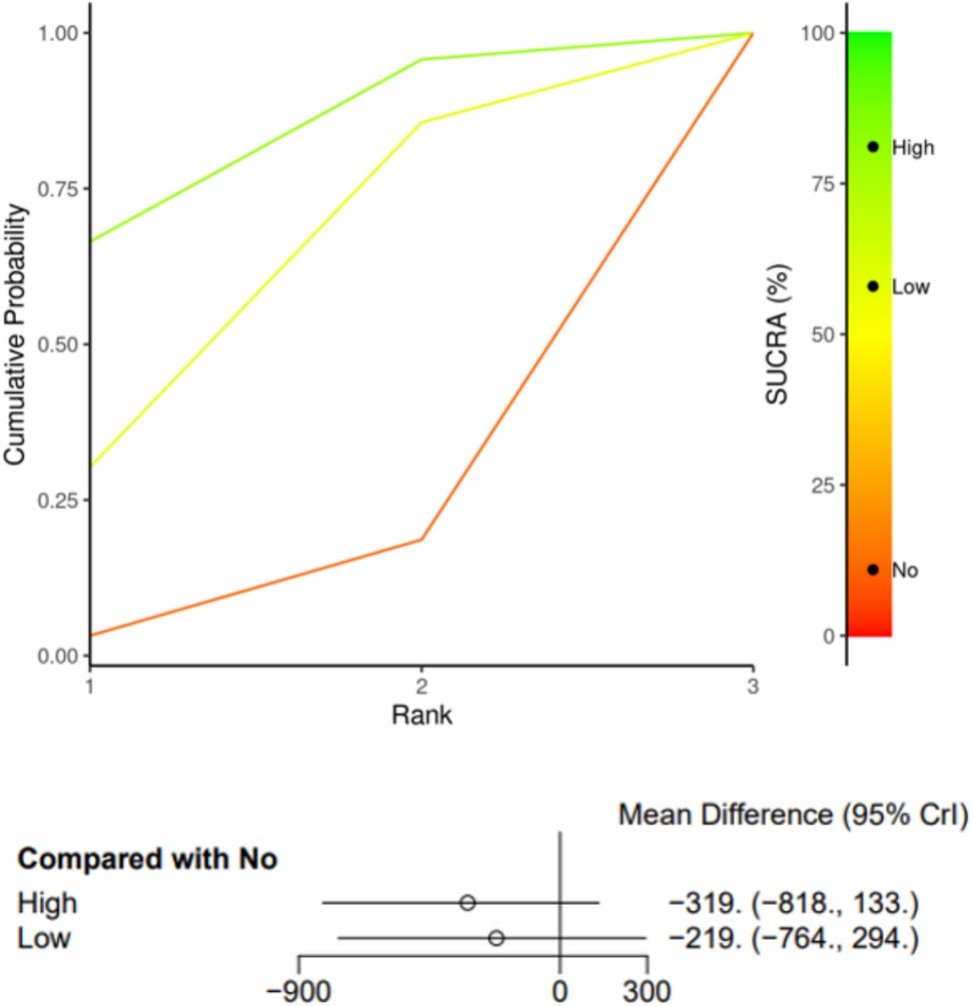
The surface under the cumulative ranking curve (SUCRA) graph (upper) and the forest plot (lower) for total estimated blood loss. High (High TXA), Low (Low TXA), No (No TXA)

None of the groups had a high probability of being the best treatment for minimizing total intraoperative EBL; however, the No TXA group (n = 147) had a high probability of being the worst treatment (81%) for minimizing total EBL (Fig. 4). Compared to the No TXA group, there was no statistically significant decrease in total EBL for the High TXA group (n = 184) (MD = −319.0 mL [−818.0 mL, 133 mL]) or the Low TXA group (n = 144) (MD = −219.0 mL [−764.0 mL, 294 mL]) (Fig. 4). Furthermore, there was no statistically significant difference in total EBL between the High TXA group and the Low TXA group (MD = −98.3 mL [−646.9 mL, 426.2 mL]). The FWA (SD) total EBL was 787.3 mL (261.5 mL), 705.3 mL (219.0 mL), and 1016.3 mL (492.2 mL) for the High TXA group, Low TXA group, and No TXA group, respectively (Table 2). However, there was inconsistency noted in the model with “very low” certainty.

**Table 2:** Outcomes for efficacy and safety for TXA by dose in this network meta-analysis

Author (year)	Groups	Patients (n)	Intraoperative EBL (mean (SD)) (mL)	Intraoperative EBL per level (mean (SD)) (mL/spinal level)	Intraoperative EBL per Hour (mean (SD)) (mL/h)	Perioperative blood transfusion (n)	Total TXA-related complications
Goobie (2018)	High Dose TXA	56	836 (373)	82 (32)	190 (73)	0	0
	No TXA	55	1031 (484)	110 (40)	230 (80)	6	0
Hasan (2021)	High Dose TXA	83	844.9 (384.9)	73.7 (29.2)	385.4 (141.2)	1	0
	Low Dose TXA	83	815.1 (383.9)	73.5 (29.2)	388.4 (150.5)	1	0
Saleh (2018)	High Dose TXA	25	181.25 (18.7)	–	84.7 (8.7) calculated from operative time (hours)	–	0
	Low Dose TXA	25	229 (22.44)	–	73.2 (7.2) calculated from operative time (hours)	–	0
	No TXA	25	266.7 (42.4)	–	76.9 (12.2) calculated from operative time (hours)	–	0
Verma (2014)	Low Dose TXA	36	783 (514)	89.0 (58.4) calculated from levels fused	–	–	0
	No TXA	47	960 (175)	106.7 (19.4) calculated from levels fused	–	–	1
Xu (2012)	High Dose TXA	20	1169.5 (270.5)	92.1 (21.3) calculated from levels fused	281.8 (65.2) calculated from operative time (hours)	–	0
	No TXA	20	2045.1 (599.3)	156.1 (45.7) calculated from levels fused	520.4 (152.5) calculated from operative time (hours)	–	0

*SD* standard deviation *TXA* tranexamic acid *EBL* estimated blood loss

### Sensitivity analysis of estimated blood loss per vertebral level by tranexamic acid dose

None of the groups had a high probability of being the best treatment for minimizing EBL per level; however, the No TXA group (n = 122) had a high probability of being the worst treatment (87%) for minimizing EBL per level (Supplemental Fig. 8). Compared to the No TXA group, there was no statistically significant decrease in EBL per level for the High TXA group (n = 159) (MD = −38.2 mL [−86.3 mL, 6.1 mL]) or the Low TXA group (n = 119) (MD = −29.5 mL [−85.2 mL, 27.3 mL]) (Supplemental Fig. 8). Furthermore, there was no statistically significant difference in EBL per level between the High TXA and the Low TXA group (MD = −8.7 mL [−66.6 mL, 45.0 mL]). The FWA (SD) EBL per level was 78.9 mL (6.3 mL), 78.2 mL (7.2 mL), and 116.3 mL (17.8 mL) for the High TXA group, Low TXA group, and No TXA group, respectively (Table 2). There was inconsistency and “very low” certainty.

### Sensitivity analysis of estimated blood loss per hour by tranexamic acid dose

None of the groups had a high probability of being the best or worst treatment for minimizing EBL per hour (Supplemental Fig. 9). Compared to the No TXA group, there was no statistically significant decrease in EBL per hour for the High TXA group (n = 184) (MD = −81.0 mL [−250.0 mL, 80.5 mL]) or the Low TXA group (n = 108) (MD = −60.2 mL [−285.0 mL, 160.0 mL]) (Supplemental Fig. 9). Furthermore, there was no statistically significant difference in EBL per hour between the High TXA group and the Low TXA group (MD = −20.7 mL [−218.2 mL, 173.5 mL]). The FWA (SD) EBL per hour was 273.8 mL (112.6 mL), 315.4 mL (133.6 mL), and 249.8 mL (150.2 mL) for the High TXA group, Low TXA group, and No TXA group, respectively (Table 2). There was no inconsistency noted with “very low” certainty.

### Transfusion rates by tranexamic acid dose

Four studies reported outcomes of perioperative blood and crystalloid transfusion [17, 18, 29, 30]. Goobie et al. (2018) found that 11% of placebo patients (n = 6/55) required perioperative packed red blood cell transfusions vs none of the 56 patients in the TXA group [17]. Only one placebo patient (and no TXA patients) received fresh-frozen plasma. Placebo patients also needed more IV crystalloid (2500 mL vs. 2,145 mL; p = 0.04). Hasan et al. (2021) reported that one patient in each of the high dose and low dose TXA groups required an allogenic blood transfusion perioperatively [18]. Crystalloid volumes were similar between groups (high dose: 1243.1 ± 303.7 mL vs. low dose: 1247.6 ± 325.3 mL; p = 0.927). Verma et al. (2014) found there was no difference in the number of patients needing perioperative transfusion between groups (TXA: n = 13/36; epsilon-aminocaproic acid: n = 11/42; saline: n = 10/47; p = 0.136) [29]. Xu et al. (2012) found no significant difference in allogenic transfusion volume between the batroxobin (B: 360 ± 263.7 mL) and TXA (C: 235 ± 163.1 mL; p = 0.069) groups [30]. However, both groups B and C had lower transfusion volme compared to the saline group (A; p < 0.0001), and a higher volume than the combined treatment group D (B vs. D: p < 0.0001; C vs. D: p = 0.006). The volume of autologous transfusion in the saline group (A: 1005.8 ± 267.5 mL) exceeded batroxobin (B: 710.7 ± 186.5 mL) and TXA (C: 634.7 ± 162.2 mL) significantly (p < 0.0001), while TXA vs. batroxobin was similar (p = 0.211). All three groups had substantially higher autologous volumes compared to group D, with reductions of 61.2%, 45.1%, and 38.5% for groups A, B and C, respectively (p < 0.0001).

### Laboratory values by tranexamic acid dose

Two studies reported changes in perioperative hemoglobin levels [17, 18]. Goobie et al. (2018) found that there was no difference in the median intraoperative hemoglobin between the saline group (9.4 [8.4–10.8]) vs. TXA (9.8 [9.2–10.8]) (p = 0.18) [17]. Hasan et al. (2021) found that there was no difference in the values of perioperative hemoglobin (high dose TXA: 13.8 ± 1.0; low dose TXA: 13.8 ± 1.1; p = 0.485) [18]. Additionally, there was no difference between the drop in perioperative hemoglobin at 48 h between the low dose vs high dose TXA groups (3.3 vs. 3.0 g/dL, respectively; p = 0.082).

### Tranexamic acid-related complications by tranexamic acid dose

There was only one TXA-related complication of uncontrolled bleeding in the No TXA group (n = 147) for an incidence of 0.7%. There were zero TXA-related complications in either the Low TXA group (n = 144) or the High TXA group (n = 184) for an incidence of 0.0% and 0.0%, respectively. Notably, there were not cases of VTE, seizures, or renal pathology in any group.

## Discussion

This NMA of five RCTs evaluates the comparative impact of high and low dose tranexamic acid regimens on intraoperative blood loss and safety outcomes in patients with undergoing PSF. We found that both high and low dose TXA regimens showed modest lower overall blood loss compared with no TXA—by roughly 200–300 mL on average—though the degree of reduction was not uniform across all metrics and did not reach statistical significance. Still, there was an 81% probability that no TXA was the worst strategy for total EBL. On a per-level basis, patients receiving TXA required approximately 30–40 mL less blood per fusion level than controls, suggesting some dose-related efficacy when accounting for construct length, although this was also not a significant finding. When blood loss was indexed to operative time, the pattern was less consistent: highdose TXA treatment showed a modest reduction in mL transfused per hour, whereas low dose TXA showed a slightly higher hourly blood loss than placebo, though this difference was not significant. Importantly, TXA—regardless of dose—was uniformly safe, with zero reported thromboembolic or neurologic complications among 328 TXA treated patients. Taken together, these findings indicate that TXA yields modest, non‐significant reductions in bleeding and appears safe, but its routine use in AIS PSF warrants further evaluation. Importantly, our analysis indicates that the certainty of evidence is very low with a small cohort size, underscoring the need to apply these findings cautiously on an individual basis until further research is available.

Perioperative blood loss in AIS patients undergoing PSF is substantially lower than in other pediatric deformities. After adjusting for weight and fusion levels, AIS cohorts report mean intraoperative EBL of 232–951 mL (≈7–15 mL/kg), whereas neuromuscular scoliosis often approaches 78% of total blood volume (25–127%)—even with antifibrinolytics, lower‐threshold EBL remains 700–1000 mL [31–33] [34, 35]. For instance, Duchenne muscular dystrophy curves can exceed 3000 mL without TXA, falling to about 1,900 mL with TXA (a 42% absolute and 58% normalized reduction) [36, 37]. Congenital and syndromic deformities show similar percentage reductions, but their baseline losses remain higher due to more extensive osteotomies and comorbidities [38–40]. This contrast highlights how AIS’s inherently lower hemorrhagic risk limits the absolute impact of antifibrinolytic therapy in this population. It is also worth noting that EBL—while the most widely reported metric—is inherently less reliable than transfusion requirements or calculated actual blood loss (ABL), both of which integrate clinical response and perioperative hemodilution. Unfortunately, transfusion thresholds and intra-operative fluid management varied across trials, and ABL data were inconsistently reported. Until standardized transfusion protocols and ABL reporting become commonplace, EBL as a proxy may be a necessary and de facto endpoint despite its recognized limitations.

Notably, high dose TXA did not confer clear advantages over low dose regimens; both arms demonstrated nearly identical mean reductions in total and per-level EBL, and the NMA probability rankings were comparable. This suggests that a lower TXA dose may suffice for most AIS PSF cases, with high dose reserved for the rare patient with exceptionally high bleeding risk or extensive osteotomies. Compared to the EBL seen in such other pediatric deformity surgeries, our study revealed much lower baseline EBLs—1016.3 mL without TXA—and more modest absolute reductions of 219.0–319.0 mL with low and high dose TXA, respectively. This corresponds to roughly a 20–31% decrease in total blood loss, far less dramatic than the 40–60% reductions seen in more complex deformity populations. Furthermore, when normalized to fused levels, AIS patients on TXA required about 30–40 mL less blood per level (versus 116.3 mL/level without TXA), whereas neuromuscular or syndromic cases often exceed 150–200 mL/level without antifibrinolytics. Thus, it appears that TXA may confer a meaningful reductions in EBL for such high-risk deformities, although its impact in AIS EBL reduction appears comparatively small, possibly reflecting the inherently lower hemorrhagic risk and simpler surgical profiles of these adolescent patients. This may be attributed to the fact that surgery for AIS typically involves fewer osteotomies and less extensive instrumentation than other pediatric spinal deformities, such as Scheuermann’s kyphosis, or adult scoliosis when performing PSF. AIS curves are generally more flexible and less rigid, so standard posterior instrumentation often suffices without extensive osteotomies [41]. The extent of spinal instrumentation in AIS is generally dictated by curve type and flexibility, with a trend toward shorter fusion constructs and selective fusion strategies to preserve segmental ROM, as opposed to the more extensive constructs often required in neuromuscular or congenital scoliosis [18, 41, 42]. Even when Ponte osteotomies are used in AIS, they are fewer in number and incur less blood loss per osteotomy than those required for Scheuermann’s kyphosis or adult scoliosis [41].

In our included RCTs, AIS patients underwent PSF with FWA operative time just above three hours and fused levels averaging 9.7–11.0, resulting in total EBLs of roughly 1,000 mL in the no‐TXA arms (mean 1016.3 mL; SD 492.2) compared with 705.3 mL (SD 219.0) and 787.3 mL (SD 261.5) in low‐ and high‐dose TXA groups, respectively. By contrast, adult spinal deformity (ASD) cases report a mean perioperative EBL ranging from 1200–2000 mL or more, with per-level blood loss often exceeding 100 mL/level, and even higher when complex osteotomies such as pedicle subtraction osteotomy are performed [43, 44]. Hidden blood loss in adults is also significant and can account for over half of total blood loss, similar to pediatric cases, but the absolute values are higher due to larger patient size, more extensive surgery, and comorbidities [44]. PSF for ASD typically takes substantially longer than PSF for AIS, with mean operative times commonly range from approximately 5 to 8.5 h, with more complex cases—such as those involving long fusions, pelvic fixation, or major osteotomies—often exceeding 7–9 h [45]. For example, Daniels et al. (2023) found that the mean operative time for ASD patients in the longest operative time group was 510 min, compared to 286 min in the lowest group, with a median around 6–7 h for typical cases [46]. In contrast, PSF for AIS is much shorter in length, with other studies reporting mean operative times generally between 3 and 3.5 h, mirroring the findings of the current study; even in instances of severe curves (Cobb angle ≥ 90°), mean operative times have been reported around 175–200 min [47–49].

The safety profile of TXA in the current study was overall reassuring. Across all dosing regimens, there were no reported adverse events related to TXA, including thromboembolic events, seizures, or renal complications. Notably, there were zero TXA-related complications in both the high dose and low dose groups. In contrast, the no-TXA group experienced a single case of uncontrolled bleeding, although this low powered finding is likely not a meaningful clinical comparison to make between groups. Overall, these findings suggest that TXA, regardless of dose, appears to be a safe adjunct in PSF for AIS, with no significant increase in complications when used perioperatively. Although concerns regarding TXA’s potential for thromboembolic and other adverse-event remain, our analysis identified no such complications in PSF for AIS, reinforcing a reassuring safety profile while highlighting the need for larger confirmation studies. It should be recognized that the randomized trials included in this review largely predate current perioperative blood-management practices. Recent real-world and registry data, such as those from the MIMO and Harms Study Group networks, report lower overall blood loss and stronger TXA effects than older RCTs. Our findings therefore reflect the limitations of historical trial data and should not be interpreted as evidence against TXA use in current practice.

This study has several limitations that must be considered when interpreting or findings. The variability in patient baseline characteristics, especially operative times, could affect transitivity, though we attempted to address this through sensitivity analysis of EBL per hour of operative time. Additionally, our analyses were likely underpowered to detect differences of the observed magnitude, as reflected by wide CIs and very low certainty. Whether continued patient accrual would ultimately yield statistical significance or instead reveal futility (e.g., via trial-sequential analysis) remains an open question. A further limitation is that most included trials reported EBL rather than directly measured or calculated blood loss. EBL is a subjective measure that can vary by local practice and documentation. Moreover, several RCTs in our network predate current blood-management pathways, which may partly explain higher EBL compared with more recent clinical series. Future studies should incorporate more objective and reproducible metrics, such as *allowable blood loss* calculations or direct quantification methods to enhance accuracy and comparability across centers. Finally, the high dose TXA group included varying dosages, which may introduce some heterogeneity. Despite these limitations, this is the first NMA focused exclusively on AIS, providing a more targeted and comprehensive analysis compared to previous meta-analyses.

## Conclusion

In this AIS-specific NMA of five RCTs, high and low dose TXA yielded only modest, non-significant reductions in total intraoperative blood loss (MD –319 to –219 mL) and per-level EBL (MD −38.2 to −29.5 mL), with wide confidence intervals reflecting low power and very low certainty of evidence. These results suggest that, while TXA may confer a small hemostatic benefit, its effect size in PSF for AIS may be limited by the inherently lower bleeding risk of this population. Crucially, no thromboembolic, neurologic, or renal complications were observed among the TXA-treated patients, reinforcing its favorable safety profile. For practicing surgeons, these data support a tailored approach: reserving TXA for cases with anticipated higher blood loss—such as longer constructs, osteotomies, or coagulopathic risk factors—rather than blanket administration. Ultimately, larger, multicenter RCTs with harmonized dosing strategies and extended follow-up are needed to clarify the optimal role of TXA in AIS and to determine whether its modest trends translate into meaningful clinical advantages.

## Supplementary Information

Below is the link to the electronic supplementary material.

Supplementary file1 (DOCX 249 kb)

## References

[1] Cheng JC, Castelein RM, Chu WC, Danielsson AJ, Dobbs MB, Grivas TB et al (2015) Adolescent idiopathic scoliosis. Nat Rev Dis Primers 1:15030. 10.1038/nrdp.2015.30 27188385 10.1038/nrdp.2015.30

[2] Force USPST, Grossman DC, Curry SJ, Owens DK, Barry MJ, Davidson KW et al (2018) Screening for adolescent idiopathic scoliosis: US preventive services task force recommendation statement. JAMA 319(2):165–172. 10.1001/jama.2017.19342 29318284 10.1001/jama.2017.19342

[3] Penha PJ, Ramos N, de Carvalho BKG, Andrade RM, Schmitt ACB, Joao SMA (2018) Prevalence of adolescent idiopathic scoliosis in the state of Sao Paulo, Brazil. Spine (Phila Pa 1976) 43(24):1710–1718. 10.1097/BRS.0000000000002725 29877996 10.1097/BRS.0000000000002725

[4] Yilmaz H, Zateri C, Kusvuran Ozkan A, Kayalar G, Berk H (2020) Prevalence of adolescent idiopathic scoliosis in Turkey: an epidemiological study. Spine J 20(6):947–955. 10.1016/j.spinee.2020.01.008 31972303 10.1016/j.spinee.2020.01.008

[5] Kuroki H (2018) Brace treatment for adolescent idiopathic scoliosis. J Clin Med. 10.3390/jcm7060136 29867010 10.3390/jcm7060136 PMC6024899

[6] Li K, Miao J, Zhang J (2021) Network meta-analysis of short-term effects of different strategies in the conservative treatment of AIS. Eur J Med Res 26(1):54. 10.1186/s40001-021-00526-6 34120641 10.1186/s40001-021-00526-6 PMC8201698

[7] Locke LL, Rhodes LN, Sheffer BW (2023) Accelerated protocols in adolescent idiopathic scoliosis surgery. Orthop Clin North Am 54(4):427–433. 10.1016/j.ocl.2023.04.003 37718082 10.1016/j.ocl.2023.04.003

[8] Tambe AD, Panikkar SJ, Millner PA, Tsirikos AI (2018) Current concepts in the surgical management of adolescent idiopathic scoliosis. Bone Joint J 100-B(4):415–424. 10.1302/0301-620X.100B4.BJJ-2017-0846.R2 29629580 10.1302/0301-620X.100B4.BJJ-2017-0846.R2

[9] Tsirikos AI, Ahuja K, Khan M (2024) Minimally invasive surgery for adolescent idiopathic scoliosis: a systematic review. J Clin Med. 10.3390/jcm13072013 38610778 10.3390/jcm13072013 PMC11012693

[10] Cheung ZB, Selverian S, Cho BH, Ball CJ, Kang-Wook Cho S (2019) Idiopathic scoliosis in children and adolescents: emerging techniques in surgical treatment. World Neurosurg 130:e737–e742. 10.1016/j.wneu.2019.06.207 31284059 10.1016/j.wneu.2019.06.207

[11] Miller DJ, Cahill PJ, Vitale MG, Shah SA (2020) Posterior correction techniques for adolescent idiopathic scoliosis. J Am Acad Orthop Surg 28(9):e363–e373. 10.5435/JAAOS-D-18-00399 31633657 10.5435/JAAOS-D-18-00399

[12] Newton PO, Marks MC, Bastrom TP, Betz R, Clements D, Lonner B et al (2013) Surgical treatment of Lenke 1 main thoracic idiopathic scoliosis: results of a prospective, multicenter study. Spine (Phila Pa 1976) 38(4):328–338. 10.1097/BRS.0b013e31826c6df4 22869062 10.1097/BRS.0b013e31826c6df4

[13] Aleid AM, Saeed HS, Aldanyowi SN, Albinsaad L, Alessa M, AlAidarous H et al (2024) Efficacy of high-dose versus low-dose tranexamic acid for reduction of blood loss in adolescent idiopathic scoliosis surgery: a systematic review and meta-analysis. Surg Neurol Int 15:473. 10.25259/SNI_644_2024 39777164 10.25259/SNI_644_2024 PMC11705159

[14] Kolz JM, Neal KM (2022) Hidden blood loss in adolescent idiopathic scoliosis surgery. Orthop Traumatol Surg Res 108(6):103216. 10.1016/j.otsr.2022.103216 35093565 10.1016/j.otsr.2022.103216

[15] Wang L, Liu J, Song X, Luo M, Chen Y (2021) Hidden blood loss in adolescent idiopathic scoliosis patients undergoing posterior spinal fusion surgery: a retrospective study of 765 cases at a single centre. BMC Musculoskelet Disord 22(1):794. 10.1186/s12891-021-04681-z 34525991 10.1186/s12891-021-04681-z PMC8444395

[16] Chen K, Wang L, Gao Q, Masood U, Zeng Z, Yang H et al (2023) Tranexamic acid can reduce blood loss in adolescent scoliosis surgery: a systematic review and meta-analysis. BMC Musculoskelet Disord 24(1):686. 10.1186/s12891-023-06811-1 37644447 10.1186/s12891-023-06811-1 PMC10463947

[17] Goobie SM, Zurakowski D, Glotzbecker MP, McCann ME, Hedequist D, Brustowicz RM et al (2018) Tranexamic acid is efficacious at decreasing the rate of blood loss in adolescent scoliosis surgery: a randomized placebo-controlled trial. J Bone Joint Surg Am 100(23):2024–2032. 10.2106/JBJS.18.00314 30516625 10.2106/JBJS.18.00314

[18] Hasan MS, Yunus SN, Ng CC, Chan CYW, Chiu CK, Kwan MK (2021) Tranexamic acid in pediatric scoliosis surgery: a prospective randomized trial comparing high-dose and low-dose tranexamic acid in adolescent idiopathic scoliosis undergoing posterior spinal fusion surgery. Spine (Phila Pa 1976) 46(22):E1170–E1177. 10.1097/BRS.0000000000004076 33882541 10.1097/BRS.0000000000004076

[19] Saleh A, Mostafa RH (2018) Increased nociception following administration of different doses of tranexamic acid in adolescent idiopathic scoliosis surgery. Open Anesth J 12:61–68. 10.2174/2589645801812010061

[20] Xiong Z, Wu K, Zhang J, Leng D, Yu Z, Zhang C et al (2020) Different dose regimens of intravenous tranexamic acid in adolescent spinal deformity surgery: a systematic review and meta-analysis. BioMed Res Int 2020:3101358. 10.1155/2020/3101358 33490241 10.1155/2020/3101358 PMC7803096

[21] Liu X, Ma Z, An J, Luo Z (2025) Comparative efficacy and safety of high-dose versus low-dose tranexamic acid in adolescent idiopathic scoliosis: a systematic review and meta-analysis. PLoS ONE 20(4):e0320391. 10.1371/journal.pone.0320391 40168355 10.1371/journal.pone.0320391 PMC11960895

[22] Hutton B, Salanti G, Caldwell DM, Chaimani A, Schmid CH, Cameron C et al (2015) The PRISMA extension statement for reporting of systematic reviews incorporating network meta-analyses of health care interventions: checklist and explanations. Ann Intern Med 162(11):777–784. 10.7326/M14-2385 26030634 10.7326/M14-2385

[23] Ouzzani M, Hammady H, Fedorowicz Z, Elmagarmid A (2016) Rayyan-a web and mobile app for systematic reviews. Syst Rev 5(1):210. 10.1186/s13643-016-0384-4 27919275 10.1186/s13643-016-0384-4 PMC5139140

[24] Higgins JP, Altman DG, Gotzsche PC, Juni P, Moher D, Oxman AD et al (2011) The Cochrane Collaboration’s tool for assessing risk of bias in randomised trials. BMJ 343:d5928. 10.1136/bmj.d5928 22008217 10.1136/bmj.d5928 PMC3196245

[25] Guyatt G, Oxman AD, Akl EA, Kunz R, Vist G, Brozek J et al (2011) GRADE guidelines: 1. introduction-GRADE evidence profiles and summary of findings tables. J Clin Epidemiol 64(4):383–394. 10.1016/j.jclinepi.2010.04.026 21195583 10.1016/j.jclinepi.2010.04.026

[26] Baumann AN, Trager RJ, Anaspure OS, Floccari L, Li Y, Baldwin KD (2024) The schroth method for pediatric scoliosis: a systematic and critical analysis review. JBJS Rev. 10.2106/JBJS.RVW.24.00096 39348476 10.2106/JBJS.RVW.24.00096

[27] Owen RK, Bradbury N, Xin Y, Cooper N, Sutton A (2019) MetaInsight: an interactive web-based tool for analyzing, interrogating, and visualizing network meta-analyses using R-shiny and netmeta. Res Synth Methods 10(4):569–581. 10.1002/jrsm.1373 31349391 10.1002/jrsm.1373 PMC6973101

[28] Nevill CR, Cooper NJ, Sutton AJ (2023) A multifaceted graphical display, including treatment ranking, was developed to aid interpretation of network meta-analysis. J Clin Epidemiol 157:83–91. 10.1016/j.jclinepi.2023.02.016 36870376 10.1016/j.jclinepi.2023.02.016

[29] Verma K, Errico T, Diefenbach C, Hoelscher C, Peters A, Dryer J et al (2014) The relative efficacy of antifibrinolytics in adolescent idiopathic scoliosis: a prospective randomized trial. J Bone Joint Surg Am 96(10):e80. 10.2106/JBJS.L.00008 24875032 10.2106/JBJS.L.00008

[30] Xu C, Wu A, Yue Y (2012) Which is more effective in adolescent idiopathic scoliosis surgery: batroxobin, tranexamic acid or a combination? Arch Orthop Trauma Surg 132(1):25–31. 10.1007/s00402-011-1390-6 21909815 10.1007/s00402-011-1390-6

[31] Jain A, Njoku DB, Sponseller PD (2012) Does patient diagnosis predict blood loss during posterior spinal fusion in children? Spine (Phila Pa 1976) 37(19):1683–1687. 10.1097/BRS.0b013e318254168f 22426452 10.1097/BRS.0b013e318254168f

[32] Jain A, Sponseller PD, Newton PO, Shah SA, Cahill PJ, Njoku DB et al (2015) Smaller body size increases the percentage of blood volume lost during posterior spinal arthrodesis. J Bone Joint Surg Am 97(6):507–511. 10.2106/JBJS.N.01104 25788308 10.2106/JBJS.N.01104

[33] Shapiro F, Sethna N (2004) Blood loss in pediatric spine surgery. Eur Spine J 13(Suppl 1):S6-17. 10.1007/s00586-004-0760-y 15316883 10.1007/s00586-004-0760-y PMC3592180

[34] Hardesty CK, Gordon ZL, Poe-Kochert C, Son-Hing JP, Thompson GH (2018) Bipolar sealer devices used in posterior spinal fusion for neuromuscular scoliosis reduce blood loss and transfusion requirements. J Pediatr Orthop 38(2):e78–e82. 10.1097/BPO.0000000000001097 29189537 10.1097/BPO.0000000000001097

[35] Kannan S, Meert KL, Mooney JF, Hillman-Wiseman C, Warrier I (2002) Bleeding and coagulation changes during spinal fusion surgery: a comparison of neuromuscular and idiopathic scoliosis patients. Pediatr Crit Care Med 3(4):364–369. 10.1097/00130478-200210000-00007 12780956 10.1097/00130478-200210000-00007

[36] Shapiro F, Zurakowski D, Sethna NF (2007) Tranexamic acid diminishes intraoperative blood loss and transfusion in spinal fusions for duchenne muscular dystrophy scoliosis. Spine (Phila Pa 1976) 32(20):2278–2283. 10.1097/BRS.0b013e31814cf139 17873823 10.1097/BRS.0b013e31814cf139

[37] Dhawale AA, Shah SA, Sponseller PD, Bastrom T, Neiss G, Yorgova P et al (2012) Are antifibrinolytics helpful in decreasing blood loss and transfusions during spinal fusion surgery in children with cerebral palsy scoliosis. Spine (Phila Pa 1976) 37(9):549–555. 10.1097/BRS.0b013e31823d009b 10.1097/BRS.0b013e31823d009b 22037532

[38] Karimi S, Lu VM, Nambiar M, Phan K, Ambikaipalan A, Mobbs RJ (2019) Antifibrinolytic agents for paediatric scoliosis surgery: a systematic review and meta-analysis. Eur Spine J 28(5):1023–1034. 10.1007/s00586-019-05911-8 30739188 10.1007/s00586-019-05911-8

[39] McNicol ED, Tzortzopoulou A, Schumann R, Carr DB, Kalra A (2016) Antifibrinolytic agents for reducing blood loss in scoliosis surgery in children. Cochrane Database Syst Rev 9(9):CD006883. 10.1002/14651858.CD006883.pub3 27643712 10.1002/14651858.CD006883.pub3 PMC6457775

[40] Yuan QM, Zhao ZH, Xu BS (2017) Efficacy and safety of tranexamic acid in reducing blood loss in scoliosis surgery: a systematic review and meta-analysis. Eur Spine J 26(1):131–139. 10.1007/s00586-016-4899-0 27900553 10.1007/s00586-016-4899-0

[41] Nasto LA, Mousavi Nasab SH, Sieczak A, Cattolico A, Ulisse P, Pola E (2024) Ponte osteotomies for treatment of spinal deformities: they are not all made equal. Eur Spine J 33(7):2787–2793. 10.1007/s00586-024-08334-2 38822151 10.1007/s00586-024-08334-2

[42] Anaspure OS, Baumann AN, Crawford MT, Davis P, Ndjonko LCM, Anari JB et al (2025) Segmental range-of-motion by vertebral level in fused and unfused patients with adolescent idiopathic scoliosis: a systematic review of the literature. Spine Deform 13(1):29–41. 10.1007/s43390-024-00978-w 39342538 10.1007/s43390-024-00978-w PMC11729208

[43] Peters A, Verma K, Slobodyanyuk K, Cheriyan T, Hoelscher C, Schwab F et al (2015) Antifibrinolytics reduce blood loss in adult spinal deformity surgery: a prospective, randomized controlled trial. Spine (Phila Pa 1976) 40(8):E443–E449. 10.1097/BRS.0000000000000799 25868100 10.1097/BRS.0000000000000799

[44] Quarto E, Bourret S, Rebollar Y, Mannem A, Cloche T, Balabaud L et al (2023) Team management in complex posterior spinal surgery allows blood loss limitation. Int Orthop 47(1):225–231. 10.1007/s00264-022-05586-9 36194284 10.1007/s00264-022-05586-9

[45] Samuel AM, Fu MC, Anandasivam NS, Webb ML, Lukasiewicz AM, Kim HJ et al (2017) After posterior fusions for adult spinal deformity, operative time is more predictive of perioperative morbidity, rather than surgical invasiveness: a need for speed? Spine (Phila Pa 1976) 42(24):1880–1887. 10.1097/BRS.0000000000002243 28538595 10.1097/BRS.0000000000002243

[46] Daniels AH, Daher M, Singh M, Balmaceno-Criss M, Lafage R, Diebo BG et al (2024) The case for operative efficiency in adult spinal deformity surgery: impact of operative time on complications, length of stay, alignment, fusion rates, and patient-reported outcomes. Spine (Phila Pa 1976) 49(5):313–320. 10.1097/BRS.0000000000004873 37942794 10.1097/BRS.0000000000004873

[47] Chan CYW, Lee SY, Ch’ng PY, Chung WH, Chiu CK, Hasan MS et al (2021) Learning curve for a dual attending surgeon strategy in posterior spinal fusion (PSF): an analysis of 105 severe adolescent idiopathic scoliosis patients (Cobb Angle >/=90 degrees). Spine (Phila Pa 1976) 46(12):E663–E670. 10.1097/BRS.0000000000003866 33306608 10.1097/BRS.0000000000003866

[48] Chiu CK, Chan CY, Aziz I, Hasan MS, Kwan MK (2016) Assessment of intraoperative blood loss at different surgical stages during posterior spinal fusion surgery in the treatment of adolescent idiopathic scoliosis. Spine (Phila Pa 1976) 41(9):E566–E573. 10.1097/BRS.0000000000001304 26630421 10.1097/BRS.0000000000001304

[49] Kwan MK, Chiu CK, Hasan MS, Tan SH, Loh LH, Yeo KS et al (2019) Perioperative outcome of single stage posterior spinal fusion for severe adolescent idiopathic scoliosis (AIS) (Cobb Angle >/=90 degrees): the role of a dual attending surgeon strategy. Spine (Phila Pa 1976) 44(6):E348–E356. 10.1097/BRS.0000000000002848 30130336 10.1097/BRS.0000000000002848

