# Supplementary material for: The efficacy and safety of high versus low doses of intravenous intraoperative tranexamic acid during spinal fusion in patients with adolescent idiopathic scoliosis: a network meta-analysis of randomized controlled trials
Source: Spine Deform. 2026 Jan 24;14(4):1077–92. doi: 10.1007/s43390-026-01289-y (PMC13323272; doi:10.1007/s43390-026-01289-y)
Supplement: Supplementary file 1 — Supplementary file1 (DOCX 249 kb) [file 43390_2026_1289_MOESM1_ESM.docx]

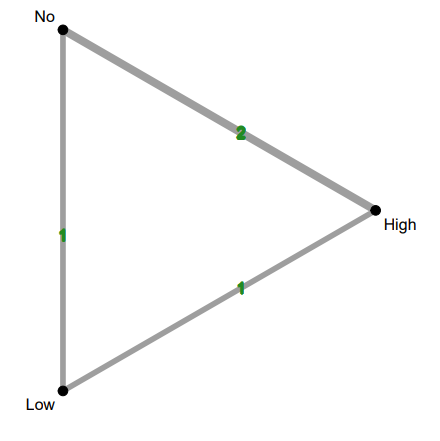


**Supplemental Figure 1:** Network geometry for estimated blood loss per level.


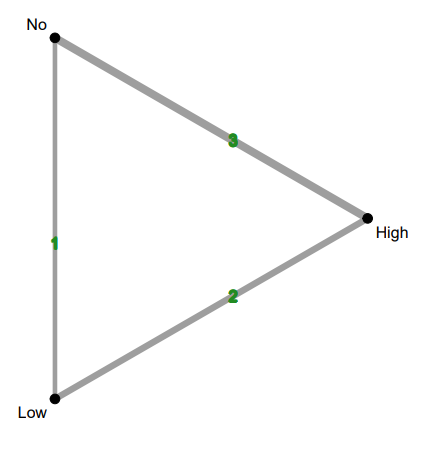


**Supplemental Figure 2**: Network geometry for estimated blood loss per hour.


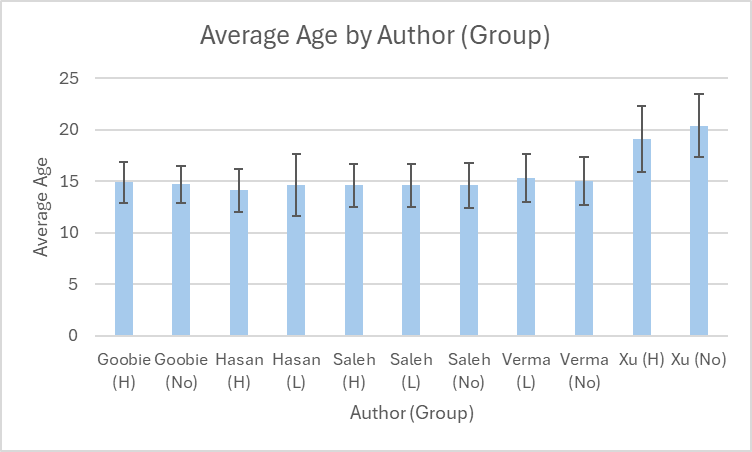


**Supplemental Figure 3**: The average age (years) by author and group for each of the included studies in this network meta-analysis. All averages here represent means. Abbreviations utilized: H, High TXA group; L, Low TXA group; No, No TXA group. Error bars on each of the bars represent one upper and lower standard deviations from the mean.


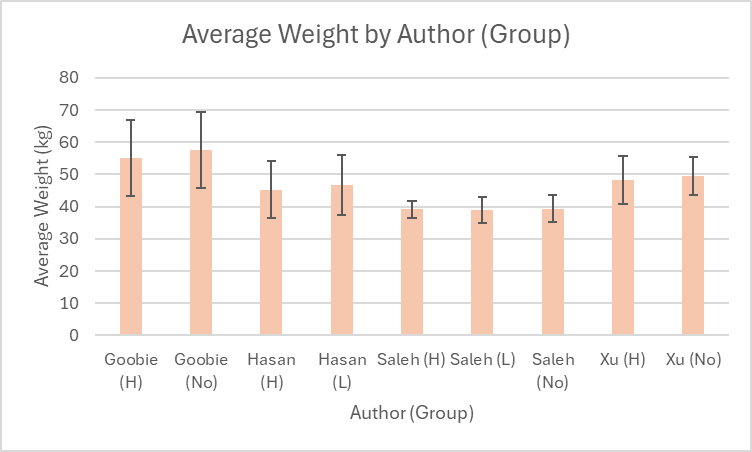


**Supplemental Figure 4**: The average weight (years) by author and group for each of the included studies in this network meta-analysis. All averages here represent means. Abbreviations utilized: H, High TXA group; L, Low TXA group; No, No TXA group. Error bars on each of the bars represent one upper and lower standard deviations from the mean.


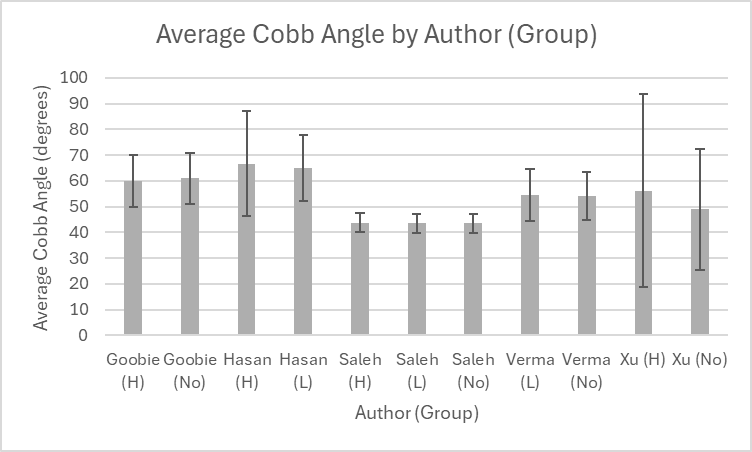


**Supplemental Figure 5**: The average Cobb Angle by author and group for each of the included studies in this network meta-analysis. All averages here represent means. Abbreviations utilized: H, High TXA group; L, Low TXA group; No, No TXA group. Error bars on each of the bars represent one upper and lower standard deviations from the mean.


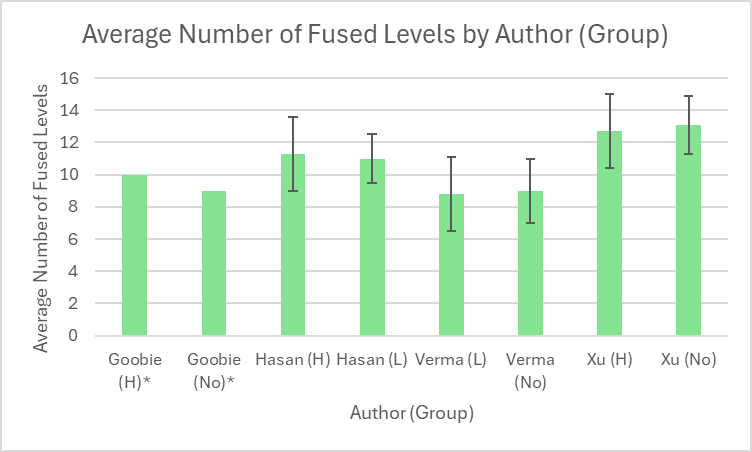


**Supplemental Figure 6**: The average fused levels by author and group for each of the included studies in this study. All averages here represent means except for those marked* (median). Abbreviations utilized: H, High TXA group; L, Low TXA group; No, No TXA group. Error bars on each bar represent one upper and lower standard deviations from the mean.


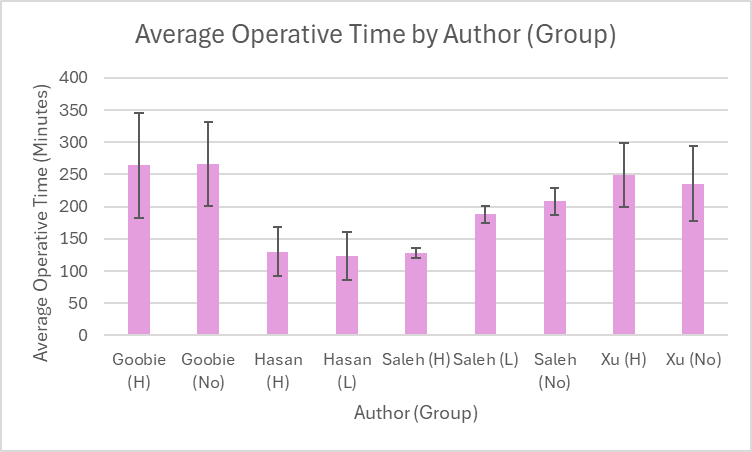


**Supplemental Figure 7**: The average operative time by author and group for each of the included studies in this study. All averages here represent means except for those marked* (median). Abbreviations utilized: H, High TXA group; L, Low TXA group; No, No TXA group. Error bars on each bar represent one upper and lower standard deviations from the mean.


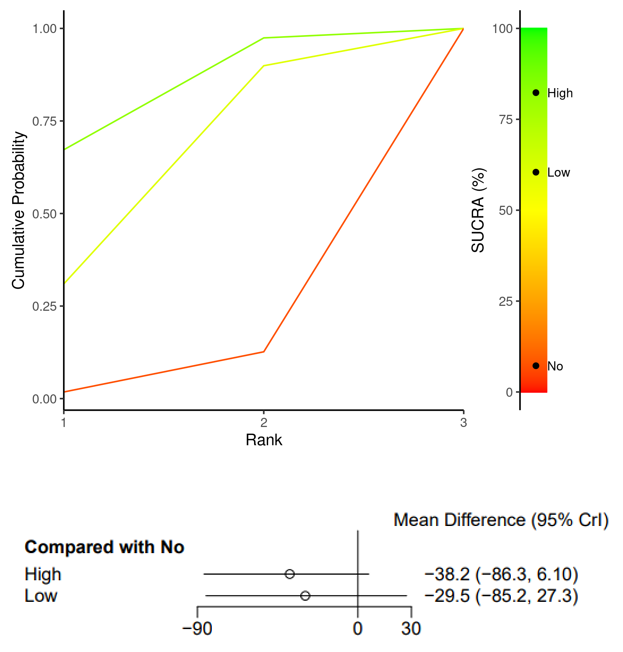


**Supplemental Figure 8:** The surface under the cumulative ranking curve (SUCRA) graph (upper) and the forest plot (lower) for estimated blood loss per level. Group abbreviations used: High (High TXA), Low (Low TXA), No (No TXA).


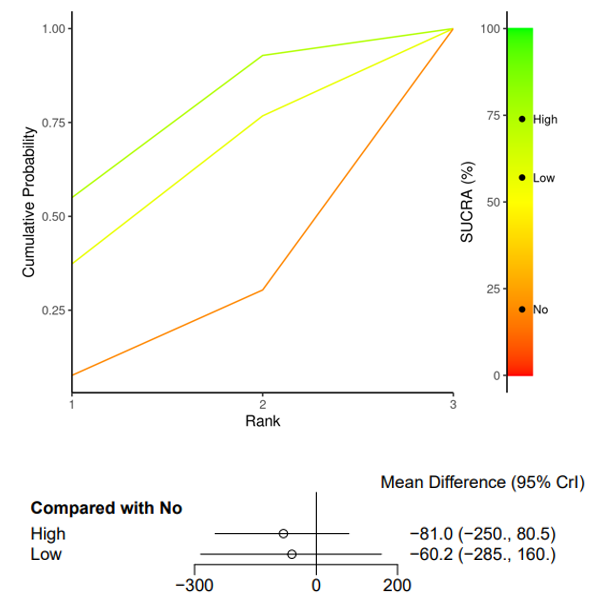


**Supplemental Figure 9:** The surface under the cumulative ranking curve (SUCRA) graph (upper) and the forest plot (lower) for estimated blood loss per hour. Group abbreviations used: High (High TXA), Low (Low TXA), No (No TXA).
